# New Transcriptome-Based SNP Markers for Noug (*Guizotia abyssinica*) and Their Conversion to KASP Markers for Population Genetics Analyses

**DOI:** 10.3390/genes11111373

**Published:** 2020-11-20

**Authors:** Sewalem Tsehay, Rodomiro Ortiz, Eva Johansson, Endashaw Bekele, Kassahun Tesfaye, Cecilia Hammenhag, Mulatu Geleta

**Affiliations:** 1 Department of Plant Breeding, Swedish University of Agricultural Sciences, P.O. Box 101, 23053 Alnarp, Sweden; rodomiro.ortiz@slu.se (R.O.); eva.johansson@slu.se (E.J.); cecilia.gustafsson@slu.se (C.H.); mulatu.geleta.dida@slu.se (M.G.); 2 Department of Microbial, Cellular and Molecular Biology, Addis Ababa University, P.O. Box 1176, Addis Ababa, Ethiopia; endashawbw@gmail.com (E.B.); kassahuntesfaye@yahoo.com (K.T.); 3 Ethiopian Biotechnology Institute, P.O. Box 5954, Addis Ababa, Ethiopia

**Keywords:** genetic diversity, genotyping, *Guizotia*, Hardy-Weinberg equilibrium, heterozygosity, KASP markers, noug, population structure, SNPs, transcriptome

## Abstract

The development and use of genomic resources are essential for understanding the population genetics of crops for their efficient conservation and enhancement. Noug (*Guizotia abyssinica*) is an economically important oilseed crop in Ethiopia and India. The present study sought to develop new DNA markers for this crop. Transcriptome sequencing was conducted on two genotypes and 628 transcript sequences containing 959 single nucleotide polymorphisms (SNPs) were developed. A competitive allele-specific PCR (KASP) assay was developed for the SNPs and used for genotyping of 24 accessions. A total of 554 loci were successfully genotyped across the accessions, and 202 polymorphic loci were used for population genetics analyses. Polymorphism information content (PIC) of the loci varied from 0.01 to 0.37 with a mean of 0.24, and about 49% of the loci showed significant deviation from the Hardy-Weinberg equilibrium. The mean expected heterozygosity was 0.27 suggesting moderately high genetic variation within accessions. Low but significant differentiation existed among accessions (FST = 0.045, *p* < 0.0001). Landrace populations from isolated areas may have useful mutations and should be conserved and used in breeding this crop. The genomic resources developed in this study were shown to be useful for population genetics research and can also be used in, e.g., association genetics.

## 1. Introduction

Noug (*Guizotia abyssinica* (L. f.) Cass.) is among the cultivated species of the family Asteraceae. Similar to other *Guizotia* species, noug is diploid (2n = 30) with relatively small chromosomes [1,2,3]. It has a larger genome size (1C = 3.8 pg) than its closely related congeners despite having the same chromosome number [4]. For example, the genome size of *G. scabra* ssp. *schimperi*, a suggested progenitor of noug, is only 55.3% of that of noug [4]. The smaller chromosomes of *G. scabra* ssp. *schimperi* when compared with that of noug [1,3] explains the larger genome size in the latter. An increase in the noug genome over its evolutionary and domestication period is likely due to genetic mechanisms such as gene duplication [5,6] and retrotransposition [7].

Noug is an underutilized but economically important minor oilseed crop mainly cultivated in Ethiopia and India. It is considered as a semi-domesticated crop with its center of origin and diversity in Ethiopia that was later introduced to India and other countries. Its cultivation outside Ethiopia and India covers countries such as Congo, Eritrea, Malawi, Sudan, Tanzania, Uganda, and Zimbabwe in Africa, and Bangladesh, Bhutan, and Nepal in Asia, as well as the Caribbean islands and the USA [8,9,10,11]. Noug has been cultivated mainly with low inputs, as it is recognized as a crop that can grow in poor and waterlogged soils vis-à-vis many other crops. Its ability to grow under poor agricultural management conditions makes it a good candidate for subsistence farming [10,11]. Within Ethiopia, the crop is cultivated at altitudes ranging from 1200 to 2700 m above sea level (masl) but the main cultivation areas are within the range of 1600 to 2200 masl [10,12]. The noug seed oil content can be as high as 50% and the oil is mainly composed of palmitic, stearic, oleic, and linoleic acids, with linoleic acid commonly accounting for more than 65% [12,13]. It has a significant dietary contribution as an important source of seed proteins, carbohydrates, minerals, vitamins, and fiber, in addition to its oil [10,14,15]. These nutrients are obtained through the consumption of whole seeds after being processed in various forms for their unique nutritional, cultural, and medicinal values [16]. These diverse local uses make it the most popular edible oilseed crop in Ethiopia.

Noug is the second-largest oil crop according to both harvest area and total production in Ethiopia, only surpassed by sesame [11]. However, its seed yield is lower than several other edible oil crops grown in the country. Traditional breeding efforts for improving seed yield and related traits have seen low successes, and only a few cultivars have been released [17]. In plant breeding programs, the selection of diverse germplasm possessing desirable characteristics and understanding differences between breeding materials are crucial steps towards cultivar development. Hence, the development and utilization of genome-wide markers are highly desirable to determine and manage genetic diversity within gene pools of crops. In line with this, expressed sequence tags (ESTs) and microsatellite (SSR) markers have been developed for noug [18], and a number of molecular marker-based studies have been conducted mainly to understand its population genetics [19,20,21,22]. However, molecular breeding has not been implemented in noug, mainly because available genomic resources and tools are highly limited. Publicly available genomic resources for noug that include expressed sequence tags (ESTs), complete chloroplast genome, and gene sequences used for phylogenetic studies can be found at the National Center for Biotechnology Information (NCBI) database (https://www.ncbi.nlm.nih.gov/nuccore/?term=Guizotia%20abyssinica).

Single nucleotide polymorphism (SNP) markers are the most recent and popular DNA markers with a diverse use in the analyses of the genomes of crops. SNP markers are amenable to high throughput genotyping by sequencing technologies and have been extensively used for genotyping of various crops for different applications, including genetic diversity analyses, genome-wide association research, and genetic linkage mapping [23,24,25,26,27,28,29,30,31,32]. However, high-throughput SNP genotyping is less suitable and not cost-effective when the number of target SNPs is in the hundreds or less. In such cases, a relatively low-cost genotyping approach, such as the Kompetitive Allele-Specific PCR (KASP) assay is preferable. SNPs converted to KASP markers can be genotyped in small labs using fluorescent resonance energy transfer (FRET)-capable plate readers and qPCR machines. SNP markers have been successfully converted to KASP markers and used for various applications in different crops, including the faba bean [33], mung bean [34], pea [35], peanut [36], rice [37,38], rye [39], sorghum [40], and wheat [41,42,43].

The objectives of the present study were (1) developing new genomic resources for noug for various applications through transcriptome sequencing of noug genotypes, SNP discovery based on the transcript sequences and converting the SNPs to KASP markers, and (2) genotyping of noug accessions using the newly developed SNP/KASP markers for population genetics analyses.

## 2. Materials and Methods

### 2.1. Plant Material

Two self-compatible noug breeding lines developed from landrace populations C19 and K13 [44] were used for transcriptome sequencing. The two self-compatible lines are significantly different from one another in several characteristics, including earliness, seed shape and size, oil content, and fatty acid composition, and hence were considered as suitable germplasm for SNP discovery. For example, K13 is characterized by early maturity, larger and shinier seeds, lower oil content, and higher oleic acid content when compared with C19. Twenty-four noug accessions grown in Ethiopia (Table S1) comprising 21 landrace populations, two released cultivars, and one breeding population (developed through crossbreeding genotypes with a high oil content) were used for genotyping using newly developed SNP/KASP markers.

### 2.2. RNA Extraction, Transcriptome Sequencing and Assembly, and SNP Calling

The two self-compatible lines were planted in a greenhouse at the Swedish University of Agricultural Sciences (SLU), Alnarp, for RNA extraction. Total RNA was separately extracted from leaf tissue of two-week-old seedlings of the two self-compatible lines using a spectrum plant total RNA kit (Sigma-Aldrich, Stockholm, Sweden). The extracted RNA was treated by Qiagen RNase free DNase (Qiagen, Stockach, Germany) to get rid of any DNA. The RNA samples were then sent to the University of California (Davis) and library preparation, transcriptome sequencing, assembly, and SNP analysis were conducted at the Genome Sciences Center. A non-normalized cDNA library was prepared following the Illumina’s guidelines and sequencing was done on the Illumina GAII, as described for the dahlia in Hodgins et al. [45]. Transcriptome assembly was done as described for Illumina sequences in Hodgins et al. [45]. A total of 4781 previously developed transcript sequences based on USDA-ARS accession PI 508077, which were functionally annotated using the Arabidopsis Information Resource (TAIR) [18] were used as a reference for SNP discovery.

After transcriptome assembly, SNP calling was made through aligning the transcript sequences to the reference sequences, and mapping the reads to the aligned sequences using BWA [46], SAMtools [47], and in-house Perl scripts. The reads of each genotype were mapped separately using the BWA aligner to generate two BAM (binary version of sequence alignment/map format) files. The SNPs between the two genotypes were determined using SAMtools. This was followed by generating a genotype table using custom-written Perl scripts in which the corresponding nucleotides of the reference transcripts are lined up with the genotype call for the two genotypes. High-quality SNPs were then obtained by filtering SNPs with at least 6× coverage. Among these, SNPs that are homozygous for both parents, having a mapped BLAST (Basic Local Alignment Search Tool) hit in the sunflower (*Helianthus annuus*), and only varying from the reference in one parent were further selected. SNPs that varied from the reference in only one parent were targeted in order to use them for genotyping of a mapping population developed using these genotypes as parents. These SNPs were further filtered by eliminating SNPs with varying sites within a 10-bp range on either side or SNPs with indels in either parent. All cases where more than one noug gene hits the same mapped *Helianthus* contig were also removed to avoid close paralogs. This filtering procedure resulted in 959 SNPs within 628 noug contigs. The accession numbers and full sequences of the 628 reference transcripts as well as the SNP sites, reference, and alternate alleles at each of 959 SNP loci are provided in Table S2.

### 2.3. Planting, Sampling, and DNA Extraction

The 24 noug accessions were planted in a greenhouse at SLU, Alnarp, for DNA extraction. A young leaf tissue was collected from two-week-old seedlings separately from individual plants for each accession using a LGC plant sample collection kit (KBS-9370-001) provided by LGC-Genomics (https://biosearch-cdn.azureedge.net/assetsv6/Plant-leaf-kit.pdf). Each accession was represented by 12 individuals except NG099 and NG108 (Table S1) that had 7 and 10 individuals, respectively. The 281 samples, representing the 24 accessions, collected into three deep-well plates (96-well), were then sent to LGC-Genomics (Hoddesdon, UK) where DNA extraction and genotyping were conducted. High-quality genomic DNA suitable for KASP genotyping was extracted using the sbeadex plant kit (https://www.biosearchtech.com/products/extraction-and-purification-reagents/dna-purification-kits/sbeadex-kits) at the LGC-Genomics facility.

### 2.4. Competitive Allele-Specific PCR (KASP) Assay Design and Genotyping

A file containing the 628 reference transcript sequences, the reference and alternate alleles of the 959 SNP loci, and their positions in the reference sequences were provided to LGC-Genomics. These data were then used for designing a competitive allele-specific PCR (KASP) assay for each SNP locus. Among the 959 SNP loci, a KASP assay was successfully designed for 931 loci, whereas 28 of them failed the KASP assay design. Hence, 931 SNP loci were used for the genotyping of the 281 samples. The genotypic data was received from LGC-genomics and analyzed using LGC’s KlusterCaller software (https://www.biosearchtech.com/support/tools/genotyping-software/klustercaller) and the genotype clusters were viewed using LGC’s SNP-viewer application (https://www.biosearchtech.com/support/tools/genotyping-software/snpviewer) that displays the two homozygotes and the heterozygote classes as separate clusters plate by plate.

### 2.5. Statistical Analyses

Various population genetics parameters were estimated using different statistical software. Genetic diversity indices were estimated for each accession or locus using Popgen32 [48], GenAlEx version 6.5 software [49], Arlequin ver 3.5 [50], and MEGA7 [51]. Pogen32 was used to calculate percent polymorphic loci and gene flow for each accession across all loci as well as the observed and effective number of alleles and allele frequency for each locus across all accessions. GenAlEx was used to calculate the Shannon information index, observed heterozygosity, standard and unbiased expected heterozygosity, and fixation indices for each accession. Arlequin was used to calculate Theta under the stepwise mutation model [52]. Tajima-Nei model [53] based estimates of average evolutionary divergence for each population were calculated using MEGA7. Arlequin was also used for the analysis of molecular variance (AMOVA) and for the Hardy-Weinberg equilibrium (HWE) test. MEGA7 was used for neighbor-joining-based cluster analysis using evolutionary distances computed based on the Tajima-Nei method [53], whereas GenAlEx was used for principal coordinate analysis (PCoA).

The program STRUCTURE (v. 2.3.4) [54] was used for population structure analysis using all loci and individuals. In this Bayesian approach-based analysis, an admixture model with a length of burn-in period of 100,000 and number of Markov chain Monte Carlo (MCMC) replications of 100,000 was used. The structure analysis was run for K = 1 to K = 15 with 20 runs at each K. The output of STRUCTURE was then used as input data for the STRUCTURESELECTOR program [55] for (1) determination of the optimal number of genetic clusters (K) using the ΔK method of Evanno et al. [56], and (2) graphical representation of the population genetic structure of the 24 accessions for the optimum K value through the application of the integrated CLUMPAK program of Kopelman et al. [57].

## 3. Results

### 3.1. The KASP Genotyping and the SNP Loci

Genotypic data across 202 SNP loci (Table S2) were used for population genetics analyses of the 24 accessions. Among the 931 SNPs targeted in the KASP genotyping, 554 were successfully genotyped across the 24 accessions, while 377 failed. Hence, the success rate of the KASP genotyping in this study is 59.5%. Of the 554 loci successfully genotyped, 286 (51.6%) were monomorphic across all individuals, whereas 268 (48.4%) were polymorphic. Among the 268 polymorphic loci, 66 of them (24.6%) had >10% missing data and were not used for further data analysis.

**Figure 1:**
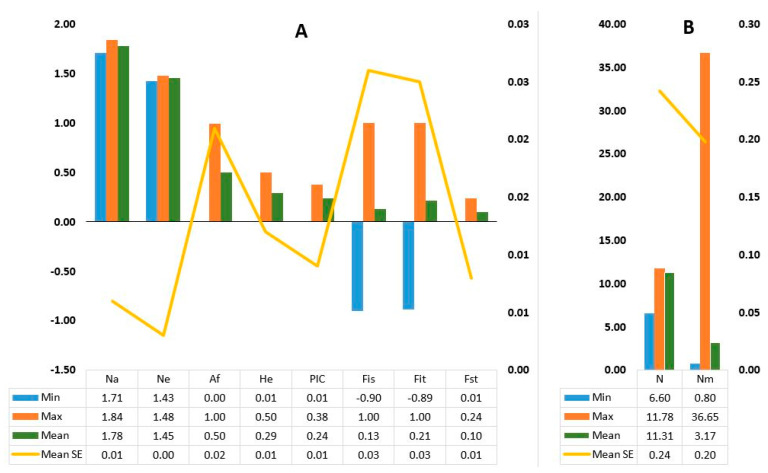
Maximum, minimum, and mean values of (**A**) observed number of alleles (Na), effective number of alleles (Ne), allele frequency (Af), expected heterozygosity (He), polymorphism information content (PIC), fixation indices (F_IS_, F_IT_ and F_ST_) per locus; (**B**) sample size (N) per population and estimate of gene-flow (Nm) per locus.

The effective number of alleles across the 202 loci varied from 1.43 to 1.48 with a mean of 1.45. Expected heterozygosity (He) varied from 0.01 to 0.50 with a mean of 0.29 whereas polymorphism information content (PIC) varied from 0.01 to 0.38 with a mean of 0.24 (Figure 1; Table S3). A large variation was observed in fixation indices among the SNP loci. The minimum, maximum, and mean values for F_IS_ were −0.90, 1.00, and 0.13, for F_IT_ were −0.89, 1.00, and 0.21, and for F_ST_ were 0.01, 0.24, and 0.10, respectively (Figure 1A). The estimate of gene flow (Nm) for each locus showed wide variation, ranging from 0.80 to 36.65, with a mean of 3.17 (Figure 1B; Table S3).

**Figure 2:**
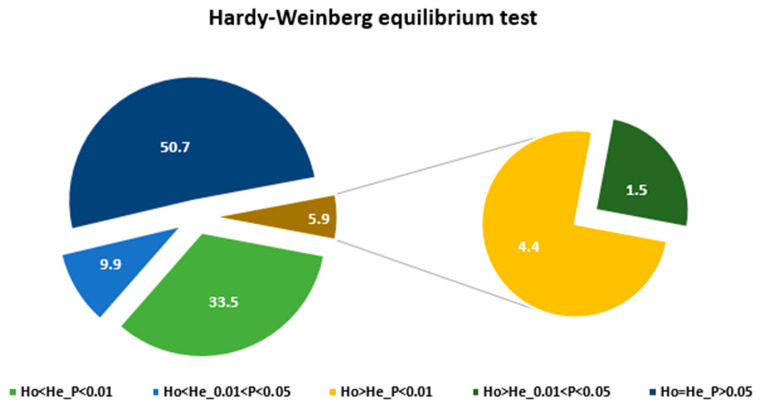
Pie-chart of the SNP loci significantly deviated from Hardy-Weinberg equilibrium (HWE) showing their proportions in terms of heterozygote excess and deficiency at different levels of significance.

The Hardy-Weinberg equilibrium (HWE) test revealed that 50.7% of the loci are at HWE whereas 49.3% of loci showed significant deviation from HWE (Figure 2; Table S3). A total of 43.4% of the loci showed heterozygote deficiency with 33.5% and 9.9% showing highly significant (*p* < 0.01) and significant (0.01 < *p* < 0.05) deviation, respectively. On the other hand, 5.9% of the loci showed excess heterozygosity with 4.4% and 1.5% showing highly significant (*p* < 0.01) and significant (0.01 < *p* < 0.05) deviation, respectively. Examples of the SNP loci showing a highly significant deviation from HWE and the description of their corresponding sunflower homologs are provided in Table 1. Twelve and four of them represent loci with heterozygote excess and deficiency, respectively. Interestingly, 10 of the 12 loci that showed heterozygote excess lacked one of the three possible genotypes expected in a bi-allelic polymorphic locus under the assumption of HWE. Similarly, all four loci with heterozygote excess lacked one of the three possible genotypes. The minor allele frequency (MAF) of the four and 12 loci ranged from 0.185 to 0.470 and 0.120 to 0.470, respectively. Among these 16 SNP loci, the change in amino acid sequences of the corresponding genes was obtained in only one locus (locus 3143A). The other 15 SNPs are synonymous substitutions (Table 1). In the case of the non-synonymous substitution, the SNP resulted in a Serine/Arginine exchange (Table 1).

### 3.2. Genetic Diversity and Population Structure

Various genetic diversity parameters were estimated for each accession based on the 202 polymorphic loci. The percent polymorphic loci (PPL) of the accessions ranged from 72% (NG108) to 85% (NG092) with a mean of 79%. The Shannon diversity index (I) ranged from 0.38 (NG124) to 0.42 (NG092) with a mean of 0.40. The lowest and highest observed (Ho) heterozygosity values were recorded for accessions NG124 (0.22) and NG099 (0.26) with a mean of 0.25. The expected heterozygosity (He) and unbiased expected heterozygosity (uHe) of the accessions ranged from 0.26 and 0.27 (NG106) to 0.28 and 0.29 (NG092) with a mean of 0.27 and 0.28, respectively. The fixation index (F) of the accessions ranged from 0.01 (NG099) to 0.16 (NG092) (Table 2). There was low variation in the Theta (H) estimated from mean heterozygosity under the stepwise mutation model [52] among the accessions with the values ranging from 1.93 (NG092) to 2.08 (NG103). The Tajima-Nei model [53] based estimates of average evolutionary divergence over sequence pairs within accessions were calculated using (1) all polymorphic loci (EAED1), (2) only polymorphic loci with an allele frequency ranging from 0.3 to 0.7 (EAED2), and (3) only polymorphic loci with minor allele frequency (MAF) of less than 0.3 (EAED3) (Table 2). The lowest values of EAED1 (0.22), EAED2 (0.38), and EAED3 (0.15) were recorded for accession NG103. The corresponding highest values were 0.31 (for accession NG086), 0.57 (for accession NG111), and 0.24 (for accession NG109), in that order. The mean values for Theta, EAED1, EAED2, and EAED3 were 2.00, 0.28, 0.48, and 0.200, respectively (Table 2).

The analysis of molecular variance (AMOVA) was conducted without grouping the accessions as well as by grouping them according to their geographic region of origin or altitudinal range of their collection sites (Table 3). The analysis showed that 95.5% of the total variation accounted for variation within accessions whereas 4.5% accounted for the variation among them (F_ST_ = 0.045, *p* < 0.0001). The vast majority of the within accession variation (93.9%) was attributed to the variation within individuals (heterozygosity). Hierarchical AMOVA was conducted by grouping 21 of the 24 accessions (excluding the two cultivars and the breeding population) into (1) three altitudinal groups, (2) six geographical regions (regions-I), and (3) two geographical regions (regions-II) (Table 3). However, only 0.12% of the total variation accounted for the variation among the altitudinal groups, which is not significant (F_CT_ = 0.001 and *p* = 0.19). Similarly, there was no significant differentiation between the six regions-I groups (F_CT_ = −0.001 and *p* = 0.67). However, there was significant differentiation between the two regions-II groups (Oromia vs. Amhara-Tigray) (F_CT_ = 0.002 and *p* = 0.047).

AMOVA-based F_ST_ was also computed for each pair of the 24 accessions, and 268 of the 276 pairs (97.1%) showed significant differentiation between them with F_ST_ values ranging from 0.017 to 0.124 (Table 4). Only eight pairs failed to show significant differentiation (F_ST_ ranging from 0.006 to 0.012). The lowest and the highest F_ST_ values were recorded for NG111 vs. NG123 and NG096 vs. NG097, respectively (Table 4). The mean F_ST_ values that reflect the average differentiation of each accession from all other accessions ranged from 0.028 (Shambu) to 0.076 (NG097). Accessions NG096 and NG124 also showed higher differentiation from the other accessions having mean F_ST_ values of 0.074 and 0.072, respectively. On the other hand, Fogera and NG123 are among the least differentiated accessions with mean F_ST_ values of 0.031 and 0.033, respectively.

**Figure 3:**
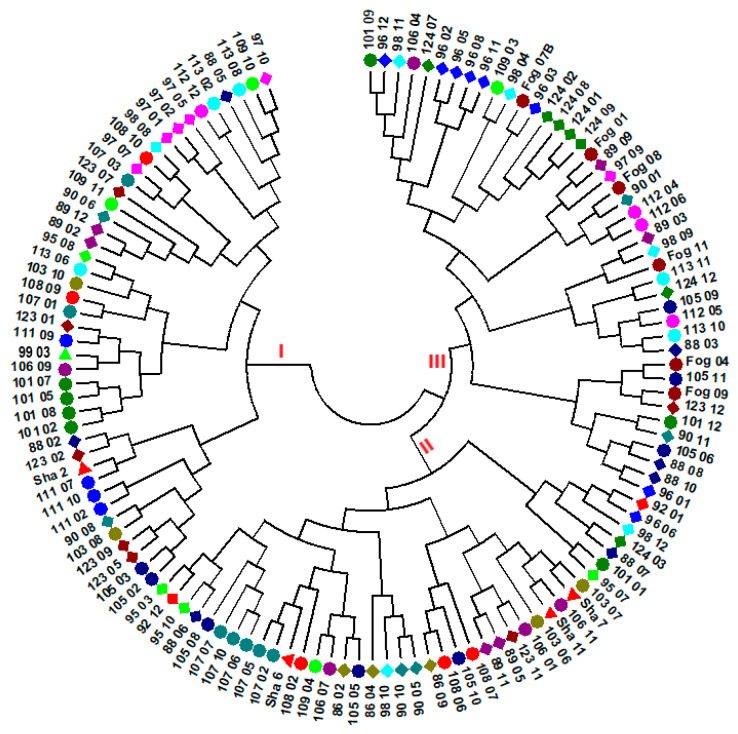
Neighbor-joining tree of 126 individuals representing the 24 accessions generated based on loci with a minor allele frequency of <0.3, using evolutionary distances computed by the Tajima-Nei method (Tajima and Nei 1984). The individual samples were coded in a way that the first two or three digits/letters represent their accessions and the last two-digit numbers represent the codes for the plant in that accession. The accession names are given without the two initial letters (NG), and Fog and Sha represent Fogera and Shambu, respectively. Individuals represented by the same shape and color belong to the same accession.

**Figure 4:**
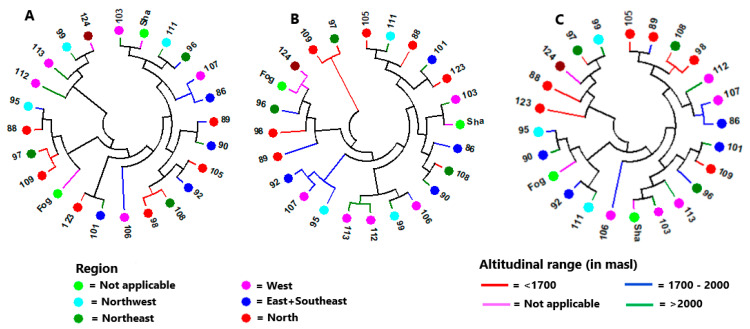
Neighbor-joining tree of the 24 accessions generated based on (**A**) all loci, (**B**) loci with a minor allele frequency of <0.3, and (**C**) loci with a minor allele frequency of ≥0.3 using evolutionary distances computed by the Tajima-Nei method [53]. The accession names are given without the two initial letters (NG) for 22 of the 24 accessions, and “Fog” and “Sha” represent Fogera and Shambu, respectively. Accessions represented by the same shape and color belong to the same region, and accessions represented with the same color tree-line belong to the same altitudinal range.

One hundred twenty-six individuals having genotypic data for all loci (no missing values) were selected across the 24 accessions and the evolutionary distance between each pair of individuals was computed for loci with MAF below 0.3 using Tajima-Nei method [53]. The neighbor-joining cluster analysis based on the evolutionary distances between the individuals resulted in three major clusters and several sub-clusters (Figure 3). Except in a few cases, individuals from the same accessions were placed in more than one cluster. For example, accession NG105 was represented by eight individuals, of which three, three, and two individuals were placed in cluster-I, II, and III, respectively. On the other hand, all six individuals of Fogera, a cultivar, were clustered in cluster-I and all four individuals of NG111 were placed in cluster-III. The Tajima-Nei method [53] based evolutionary distance was also used for neighbor-joining cluster analysis at the accession level using three sets of loci: all polymorphic loci, only loci with MAF of <0.3, and only loci with MAF of ≥0.3 (Figure 4). As depicted in Figure 4A–C, the clustering patterns of the accessions are clearly different among the three data sets. The analysis revealed that the clustering patterns of the accessions according to their geographic regions or altitudinal range of origin were poorly defined.

**Figure 5:**
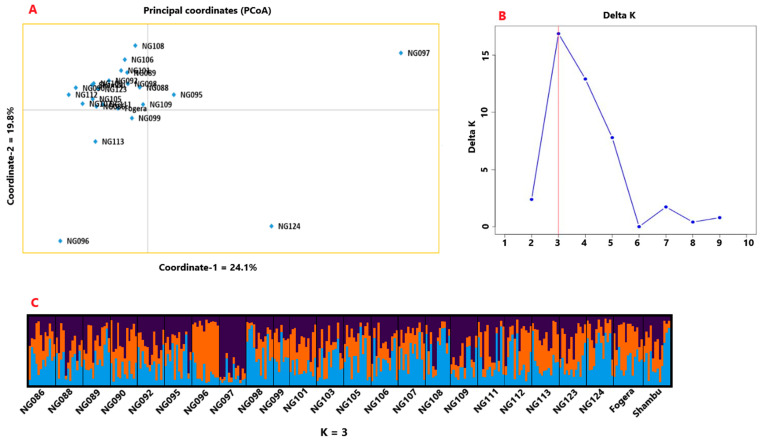
(**A**) Principal coordinate analysis (PCoA) based two-dimensional plot for the 24 accessions, in which the first and the second axes explained 24% and 20% of the total variation, respectively, (**B**) ΔK plot showing its maximum value at K = 3 suggesting the optimal number of genetic clusters (populations) of three, and (**C**) graphical representation of the population genetic structure of the 24 accessions for K = 3. The three colors represent the three clusters and the proportion of each color in each accession represents the average proportion of the alleles that placed each accession under the three clusters.

Principal coordinate analysis (PCoA) was also conducted to determine the relationship between the accessions. In the two-dimensional plot generated, the first and the second coordinates explained 24% and 20% of the total variation among the accessions (Figure 5A). This analysis revealed that most of the accessions were tightly clustered together suggesting low differentiation among them. However, accessions NG097, NG124, and NG096 were clearly separated from the other accessions in this two-dimensional plot.

The admixture model-based population genetic structure analysis conducted using STRUCTURE [54] and STRUCTURESELECTOR programs [55] revealed that the optimal number of genetic clusters (K) is three, as per the ΔK method of Evanno et al. [56] (Figure 5B). Hence, the 281 individuals representing the 24 accessions were reduced to three genetic populations. The graphical representation of the population genetic structure of the 24 accessions at K = 3, clearly showed that all accessions have alleles that originated from the three clusters (genetic populations), thus suggesting low differentiation between the accessions. In line with the results of the PCoA, accessions NG096, NG097, and NG124 showed higher differentiation than the other accessions in this analysis. The alleles of NG096, NG097, and NG124 were predominantly originated from cluster-2 (orange), cluster-3 (purple), and cluster-1 (blue), respectively (Figure 5C).

## 4. Discussion

The present study used transcriptome sequencing for the development of novel SNP markers for noug, which were used to design KASP assays and for genotyping 24 Ethiopian noug accessions. The fact that transcript sequences of only two genotypes were used for SNP discovery and the multi-step stringent procedures followed to identify and filter the SNP markers led to a relatively small number of SNPs (959). The use of a few genotypes as an SNP discovery panel can lead to an ascertainment bias [58,59]. This means that our SNP discovery approach may have left rare alleles in noug gene pool corresponding to the target transcripts undiscovered. However, the 202 polymorphic loci used in this study included loci with minor allele frequency (MAF) ranging from below 1% (rare alleles) to almost 50%, and hence the SNP discovery approach is not expected to affect the results of population genetics analyses. Although 97% of these SNPs passed the KASP assay design, only 59.5% of them were successfully used for genotyping the 24 accessions. In other words, 377 SNPs that passed the assay design (40.5%) failed at the genotyping stage. The failure could be because the SNPs do not exist in the germplasm targeted for genotyping or the primers failed to anneal to the target sequences due to sequence variation. In this study, only bi-allelic SNPs developed based on the two genotypes were used. If such SNPs are restricted to a small subset of the crop’s gene pool, it is highly likely that they fail (including the case when a nucleotide at SNP locus is different from both alleles) when applied to a diverse germplasm. Further research through resequencing of the target regions using Sanger sequencing or targeted genotyping by sequencing methods, such as SeqSNP (LGC Genomics) will shed light on the factors behind the failure.

Genetic polymorphism research remains key for the understanding of variability in organisms’ genetic makeup, and it is usually a prerequisite for the analysis of genetic variation for use in conservation and practical plant breeding. The development of genomic resources and tools for a crop is a crucial step both for the determination of genetic diversity and for the development of DNA markers associated with desirable traits for use in marker-aided breeding, and such resources and tools are often lacking for underutilized and minor crops. In the present study, useful and novel genetic information was gathered for noug despite the moderate success rate of the KASP genotyping.

### 4.1. The SNP/KASP Markers

SNP loci can be bi-, tri-, or tetra-allelic [60,61] but bi-allelic SNPs are the most commonly used because of their abundance and simplicity for use in genetic analyses. However, the level of polymorphism of bi-allelic SNPs may be lower, on average, when compared with other types of SNPs. Similar to other types of molecular markers, transcriptome-based SNPs are generally less polymorphic than non-genic SNPs. In this study, only 48.4% of the genotyped loci were polymorphic, which is not surprising as the SNPs are located in the exons of their corresponding genes and a limited number of accessions were analyzed. Polymorphism information content (PIC) and heterozygosity are common measures of polymorphism of a marker locus [62,63]. In the present study, the PIC of each SNP locus was calculated according to Hildebrand et al. [62]. In this approach, the maximum PIC value for bi-allelic SNPs is 0.375, and it is obtained when both alleles had a frequency of 0.5. The SNPs used in this study are bi-allelic, and their PIC values ranged from 0.01 to 0.37 with a mean of 0.24. Fifty percent of these SNP loci have a PIC value of more than 0.25 and hence are highly informative, and can be prioritized for various applications including for genetic diversity analysis of wider noug genetic resources and its weedy/wild close relatives. Two SNP-based studies in rice cultivars also reported a similar range in PIC values with a mean of 0.23 [64] and 0.28 [65].

In this study, 30% of the SNP loci have a minor allele frequency of less than 0.1, and as a result, the overall mean effective number of alleles was slightly below 1.5. The average observed (Ho) and expected (He) heterozygosities across the 202 loci were 0.25 and 0.29, respectively. In a study on a single noug population conducted using 43 EST-SSR markers, Dempewolf et al. [18] reported Ho and He values of 0.49 and 0.54, respectively. A separate study using a subset of these EST-SSR markers in 29 noug populations resulted in slightly lower values (Ho = 0.40 and He = 46) [22]. The average Nei’s gene diversity (Hs), an equivalent of expected heterozygosity, estimated based on random amplified polymorphic DNA (RAPD) markers [19] and amplified fragment length polymorphism (AFLP) markers [20] were 0.18 and 0.21, respectively. Similar levels of variation were obtained in *Guizotia scabra*, a closely related wild/weedy species [66]. These values are a bit lower than values obtained in the present study although the total number of alleles in the RAPD and AFLP based studies were 376 and 966, respectively [19,20]. This is partly because both RAPD and AFLP are dominant markers that generally underestimate the polymorphism level of a marker locus. The results reflect the advantage of co-dominant markers, such as SNPs and SSRs over dominant markers as have been reported in various previous publications [67,68,69]. On the other hand, the higher genetic variation reported in an EST-SSR (multi-allelic)-based study in noug [22] when compared to the present study (bi-allelic SNPs) is likely because of a higher effective number of alleles per locus (2.2) and a larger sample size of the former.

The fixation indices (F_IT_, F_IS_, and F_ST_), also referred to as F-statistics, are measures of inbreeding in terms of total population (T), sub-populations (S), and individuals (I) for each locus [70,71]. All three indices have a maximum value of one. Negative values of F_IS_ and F_IT_ indicate excess heterozygosity and a value of one for these indices indicates 100% homozygosity in each sub-population. For F_ST_, a measure of differentiation of sub-populations, the maximum value is attained when sub-populations are fixed for different alleles. In the present study, each accession is considered as a sub-population and the 24 accessions together form a total population. Noug is a strictly outcrossing species [11,44,72], and hence the overall observed heterozygosity (Ho) is expected to be higher than expected heterozygosity (He) if all other HWE assumptions are met. However, the mean Ho was less than the mean He for the polymorphic loci in the present study (Table 2; Table S3). Similarly, Ho was less than He, on average, in an EST-SSR-based study in noug [18,22]. Large variation was observed in fixation indices among the SNP loci. For example, F_IS_ values ranged from −0.90 to 1.00, indicating that some loci are in a state of heterozygote excess whereas some other loci lack heterozygotes. The mean values of F_IT_, F_IS_, and F_ST_ revealed in the present study were 0.13, 0.21, and 0.10 in that order. The values of these indices were 0.19, 0.15, and 0.04 in an EST-SSR based study [22]. The results suggest the overall low but significant heterozygote deficiency and low population differentiation in noug. The HWE test in this study revealed that about half of the loci (49.3%) showed significant deviation from HWE (Figure 2; Table S3). Interestingly, the vast majority of these loci (88%) showed heterozygote deficiency and only 12% showed heterozygote excess. The result clearly showed that different loci are under different kinds and levels of selection pressure, and other evolutionary forces, with overall heterozygote disadvantage in noug. Given that the study is based on genic SNPs, such a result is not unexpected.

Considering the 16 loci with a highly significant deviation from HWE (Table 1), 10 of the 12 loci that showed heterozygote excess lacked one of the two homozygous genotypes although their minor allele frequency (MAF) was as high as 0.47. This suggests that one of the two alleles in each locus makes the homozygous genotypes less fit, or the locus is in linkage disequilibrium (LD) with other locus or loci with a significant fitness value within its corresponding gene or other genes nearby. Among these loci, 3143A and 3143B are within the coding region of the 17.8 kDa class I heat shock protein-like gene. The SNP at the locus 3143A resulted in a Serine/Arginine non-synonymous substitution of the 99th amino acid of the protein coded by this gene. The 17.8 kDa class I heat shock protein is one of the small heat shock proteins in plants that are produced in response to high temperature stress [73]. Hence, further analysis may reveal variation in the response to heat stress among different genotypes of this locus. The SNPs that resulted in synonymous substitution are likely in LD with other locus or loci that affect the fitness of individual genotypes. Similarly, all four loci with heterozygote deficiency (Table 1) lacked heterozygous genotype despite having an MAF ranging from 0.19 to 0.47. However, all four resulted in synonymous amino acid substitution suggesting that these loci are tightly linked to a locus where strong heterozygote disadvantage is manifested. Detailed studies on the genes harboring these 16 SNPs using diverse noug germplasm may shed light on their functions.

### 4.2. Genetic Variation within Accessions

The 24 accessions used in the present study showed a relatively narrow range of variation in terms of percent polymorphic loci (PPL). The accessions with the lowest (NG108, 72%) and highest PPL (NG092, 85%) were from northeastern and eastern Ethiopia, respectively. Interestingly, both accessions are from areas less known for noug cultivation. In terms of genetic variation within accessions (I, He, and uHe), NG108 and NG106 are the lowest whereas NG092 and NG095 are the highest. These landrace populations are from different regions and hence the levels of genetic variation within landrace populations cannot be attributed to regions of cultivation. Similarly, NG092, NG095, and NG106 are landrace populations collected from middle-altitudes (Table S1), and the mean values of I, He, and uHe are similar for the three altitudinal groups, and hence altitude does not seem to have a significant effect on the extent of genetic variation. The estimates of genetic variation within accessions (I, He, and uHe) for the two cultivars (Fogera and Shambu), are close to the overall mean values of each parameter. Noug breeding in Ethiopia involves mass and recurrent selection [17]. Seemingly, the breeding methods used did not significantly affect the genetic variation of these cultivars. It could also be the case that the genetic variation in these cultivars has been increased (after their release) as a result of unintended gene flow from landraces grown in adjacent areas during seed multiplication or regeneration of the cultivars by the Ethiopian Institute of Agricultural Research at their field sites.

The 24 accessions showed several folds of variation in terms of fixation index (F) ranging from 0.013 (NG099) to 0.162 (NG092). Accession NG092 differs from most of the accessions not only in its higher genetic variation but also in having a larger deviation from HWE on average. On the other hand, the accessions are quite similar in terms of showing a low level of variation in diversity parameter Theta (H) that measures nucleotide diversity based on effective population size and mutation rate [52]. The similarly low Theta (H) values for the accessions are related to the case that they have a small variation in PPL, similar effective population size, and each accession has only two alleles at a polymorphic locus (bi-allelic SNPs).

Analysis of average evolutionary divergence over sequence pairs within populations using the Tajima-Nei model [53] produced interesting results. In order to evaluate the effect of allele frequencies in estimating evolutionary divergence between individuals within populations, this analysis was separately conducted for loci with MAF equal to or above 0.3 (EAED_2_) and loci with MAF below 0.3 (EAED_3_), in addition to their combined analysis (EAED_1_). A slightly higher positive correlation (r = 0.79) for EAED_1_ vs. EAED_2_ than for EAED_1_ vs. EAED_3_ (r = 0.75) suggests that loci with MAF equal to or above 0.3 may have a higher effect on overall evolutionary divergence within populations than loci with a MAF below 0.3. The lack of correlation between EAED_2_ and EAED_3_ suggests that the selection of noug landrace populations for conservation and breeding should consider SNP loci with a MAF below 0.3 and equal to or above 0.3 separately, as different populations may be more valuable for conserving higher genetic diversity or in harboring desirable traits in the case of the two groups of loci. For example, accession NG111 is the highest in EAED_2_ but the second-lowest in EAED_3_, revealing that it has lower frequencies for MAF below 0.3 when compared to almost all other accessions. This is different from the case in accession NG103, where all three EAEDs are the lowest.

Noug accessions from low altitudes areas (1400 to 1680 masl) had the lowest EAED_2_ (0.447) and highest EAED_3_ (0.205) compared to the other two altitudinal areas. The results suggest that low-frequency alleles are more common in the lowland than in the highland areas of noug cultivation. This provides a slightly higher weight for lowland areas as sources of new alleles than higher altitude areas. The comparison of the two cultivars and the breeding population on one hand and the landrace populations on the hand clearly indicate that the breeding process did not have negative effects on the overall genetic divergence between individuals with populations, including for loci with a MAF below 0.3. This is probably because the loci included in the present study do not have an effect on the traits targeted for breeding and hence the alleles were not differentially selected.

### 4.3. Genetic Variation among Accessions and Population Structure

Partitioning the total genetic variation into the within and among populations components is an important step in understanding the genetic structure of populations and their adaptation to local environmental conditions. Outcrossing species generally tend to have higher genetic variation within populations than among populations, which is partly attributable to higher gene flow between populations than in the case of self-pollinating species [74,75]. Both the present study and previous DNA marker-based studies in noug [19,20] revealed higher genetic variation within populations than among populations. In the RAPD [19] and AFLP [20] based studies, about 35% and 23%, respectively, of the total variation accounted for population differentiation. In the present study, however, only 4.5% of the total variation differentiated the populations. The significantly lower population differentiation in the present study is mainly because it is based on transcriptome derived bi-allelic SNP markers that are generally more conserved than RAPD and AFLP-based markers.

In an EST-SSR-based study, Dempewolf et al. [22] reported a significant population differentiation accounting for 6% of the total variation, which is quite similar to the result of the present study. An EST-SSR based study in the Ethiopian potato (*Plectranthus edulis*), an outcrossing species, and a clonally propagating crop, also revealed very low genetic differentiation (2.5%) among populations [76]. Generally, outcrossing species have lower variation among populations than predominately self-pollinating species, as revealed using different marker systems in crops such as sorghum (83% [77,78]; 70% [79,80]), common beans (95% [81]), field peas (41% [82]), and durum wheat (31% [25]). However, it should be noted that self-pollinating species can have a low population differentiation when factors other than the mating system affecting the population structure are strong. For example, variation among populations accounted for only 13% and 6% of the total variation in arabica coffee [83] and korarima [84], respectively.

In agreement with previous noug research [19,20], there was no significant differentiation between the three altitudinal groups of accessions in the present study, as revealed by hierarchical AMOVA. However, alleles specific to each altitudinal group were present (Table S4). Four, three, and one specific allele(s) were recorded for low (1400 to 1680 masl), middle (1820 to 1968 masl) and high (2045 to 2590 masl) altitudinal groups. It is interesting to note the presence of more specific alleles in low-altitude areas than in high-altitude areas despite the fact that noug cultivation is more prominent in high-altitude areas. The result suggests the importance of including low-altitude populations in the noug breeding program. Similarly, there was no significant differentiation between the six regional groups of accessions although the grouping was made under the assumption that higher access to gene flow exists within the groups than among the groups. The marginally significant differentiation between accessions from the Oromia region and Amhara-Tigray region may suggest a slightly higher level of germplasm exchange within the regions than the nation-wide average.

The cluster analysis at the individual genotype level produced three major clusters (Figure 3). However, there was no clear clustering pattern of genotypes according to their accession, which is in line with the low population differentiation obtained through AMOVA. The lack of significant differentiation among altitudinal and regional groups revealed through AMOVA was also evident in the cluster analysis at the level of accessions (Figure 4). This is the case even when only loci with a MAF below 0.3 (loci close to fixation) was used, suggesting a wide distribution of low-frequency alleles. In the PCoA that explained 44% of the total variation in its first two principal axes, the accessions were tightly clustered together with the exception of NG096, NG097, and NG124. Accession NG097 has the highest mean pair-wise F_ST_ (0.076) followed by NG096 (0.074; Table 4). Accession NG097 lacked alleles at two loci that exist in all other landrace accessions (loci 3926 and 12963A). It also lacked a number of alleles present in the vast majority of accessions. Similarly, NG096 lacked alleles common to most accessions at a number of loci. Unlike other landrace accessions, these two accessions were collected from areas where noug cultivation is rare, and their higher genetic differentiation could be the result of isolation by distance [85] by being kept at a household/local community-level for a long time. Accession NG124 is a breeding population developed through crossbreeding of selected genotypes from different landrace populations for improved oil and oleic acid content. Unlike all other accessions, it lacks an allele at a locus (locus 1117) within a gene that codes for tobamovirus multiplication protein 1-like protein. Tobamovirus multiplication protein 1 is required for efficient multiplication of a tobamovirus in *Arabidopsis* [86,87]. Further research on this locus is necessary to determine its function in noug.

Various approaches have been used to determine the source of individual genotypes in population genetics research [54,88,89,90]. Pritchard et al. [54] developed a model-based method of population structure analysis for multi-locus genotypic data, in which populations that are characterized by a set of allele frequencies across loci are assumed. In this approach, individual members of predefined populations are probabilistically assigned to a single cluster (inferred population) or receive joint assignment to more than one cluster if the model determines that they are admixed. In this study, we used 24 predefined populations (accessions) to determine the population genetic structure in noug using this model. The analysis using the ΔK method of Evanno et al. [56] showed that the individuals within these predefined populations most likely originated from three genetic populations (K = 3). Interestingly, all individuals across the predefined populations are the results of admixture from the three genetic populations although it is to a different extent (ranging from 13 to 55% on average, data not shown). This and other points discussed above generally show weak population structure in noug due to population admixture caused by strong gene flow between populations via pollen and a step by step nation-wide germplasm exchange.

## 5. Conclusions

In the present study, transcriptome sequencing was conducted, and novel SNP markers were identified in noug. More than 50% of these SNPs were successfully converted to KASP markers and used for the genotyping of noug populations collected from wide geographic areas in Ethiopia. Polymorphic SNP/KASP markers were used for population genetics analyses. The loci with high PIC or showing highly significant deviation from HWE should be prioritized for further research in this crop. The study revealed low but significant differentiation between noug populations. Nonetheless, there was generally a lack of, or poor differentiation between noug at different altitudinal ranges or regions level. This indicates strong gene flow between populations grown at different altitudes and regions, particularly between major noug cultivation areas. However, this study also gave clear indications that landrace populations cultivated in isolated areas deviate in genetic diversity from those populations in common noug cultivation areas. Thus, such populations from isolated areas in Ethiopia may be sources of useful mutations, and hence should be considered for their conservation and use in breeding of this oil crop. Overall, the transcript sequences and the SNP and KASP markers developed in this study are highly useful resources for applications such as population genetics analyses and genome-wide association research. However, the markers are relatively small in number and were developed based on only two genotypes. Hence, methods such as RNAseq based on a larger number of diverse germplasm should be applied to develop DNA markers, in the thousands, that can have wide applications by avoiding potential drawbacks associated with ascertainment bias. This approach facilitates the identification of markers associated with traits of interest through genetic linkage and association mapping, for their use in marker-aided breeding.

## Figures and Tables

**Table 1:** Description of polymorphic single nucleotide polymorphic (SNP) loci that showed highly significant deviation from Hardy-Weinberg Equilibrium (HWE), and corresponding sunflower (*Helianthus annuus*) homologs of noug genes harboring the SNPs.

Noug Contig	SNP Locus	SNP PINC ^c^	Ref_Alt ^d,e^	Missing Genotype	MAF ^f^	*Helianthus annuus* Homologue: Accession Number and Aligned Region
CL3143Contig1	3143A ^a^	376	G_C	GG	0.131	17.8 kDa class I heat shock protein-like_LOC110904834: XM_022150704; 212..484
	3143B ^a^	388	C_A	AA	0.120	
CCHT13019.b1F16.ab1	13,019A ^a^	30	C_A	AA	0.389	Two-component response regulator-like PRR73_LOC110868813: XM_022118076; 1133..1903
	13,019C ^b^	372	T_A	AT	0.448	
CCHT3719.b1M18.ab1	3719A ^a^	502	A_G	AA	0.239	TPR2-like protein_LOC110930065: XM_022173292; 2716..3466
CCHT4593.b1B22.ab1	4593B ^a^	274	A_C	None	0.389	Calnexin homolog_LOC110865890: XM_022115225; 1115..1583
CCHT4736.b1P07.ab1	4736 ^a^	498	G_C	GG	0.293	Cytochrome P450 CYP82D47-like_LOC110879526: XM_022127994; 1210..1778
CCHT7954.b1C22.ab1	7954A ^a^	387	T_C	CC	0.371	Uncharacterized protein_LOC110878690: XM_022127042; 145..855
CCHT8585.b1B11.ab1	8585 ^a^	280	C_T	CC	0.149	Uncharacterized protein_ LOC110927216: XM_022170855; 1193..1751
CCHT10160.b1P19.ab1	10,160 ^a^	328	G_C	GG	0.441	UDP-arabinopyranose mutase 1-like_ LOC110915474: XM_022160180; 62..679
CCHT13180.b1H07.ab1	13,180 ^a^	460	G_A	None	0.432	40S ribosomal protein S13_LOC110937753: XM_022180210; 4..544
CCHT17789.b1J07.ab1	17,789 ^a^	698	C_A	CC	0.245	Uncharacterized 38.1 kDa protein-like_LOC110867109: XM_022116245; 208..521
CCHT20996.b1G18.ab1	20,996B ^a^	636	C_T	CC	0.470	Heat shock 70 kDa protein 14 like_LOC110894223: XM_022141417, 93..831
CCHT17807.b1N11.ab1	17,807 ^b^	350	T_G	GT	0.250	Nucleobase-ascorbate transporter 6-like_LOC110941756: XM_022183418; 123..833
CCHT17571.b1F02.ab1	17,571 ^b^	408	T_A	AT	0.470	Probable ADP-ribosylation factor GTPase-activating protein AGD14_LOC110886873: XM_022134739; 92..748
CCHT4779.b1F19.ab1	4779 ^b^	255	A_C	AC	0.185	Probable E3 ubiquitin-protein ligase ARI1_LOC110929292: XM_022172423; 1130..1867

^a^ = heterozygote excess; ^b^ = heterozygote deficit. ^c^ SNP PINC = SNP position in noug contig. ^d^ Ref_Alt = Reference allele_Alternate allele; ^e^ All SNPs resulted in synonymous amino acid substitution except SNP at locus 3143A that led to the non-synonymous substitution of Serine vs. Arginine, at position 99 of the amino acid sequence of the 17.8 kDa class I heat shock protein-like protein; ^f^ MAF = minor allele frequency.

**Table 2:** Summary of genetic diversity estimates for 24 noug accessions based on 202 single-nucleotide polymorphic (SNP) loci or its subsets, and mean values for different groups and all accessions.

Acc	PPL	I	Ho	He	uHe	F	Theta	EAED_1_	EAED_2_	EAED_3_
NG086 ^a^	75.7	0.391	0.238	0.262	0.274	0.105	2.016	0.313	0.554	0.207
NG088 ^a^	77.7	0.396	0.242	0.263	0.276	0.092	2.004	0.276	0.468	0.198
NG089 ^a^	75.7	0.387	0.248	0.258	0.270	0.042	2.038	0.294	0.528	0.201
NG090 ^a^	80.1	0.403	0.244	0.268	0.280	0.084	1.978	0.274	0.489	0.187
NG092 ^a^	84.0	0.419	0.235	0.278	0.290	0.165	1.927	0.304	0.541	0.206
NG095 ^a^	80.6	0.415	0.256	0.277	0.289	0.087	1.934	0.285	0.497	0.196
NG096 ^a^	73.8	0.381	0.259	0.255	0.267	0.017	2.057	0.257	0.454	0.176
NG097 ^a^	76.7	0.384	0.224	0.254	0.266	0.122	2.063	0.256	0.430	0.183
NG098 ^a^	78.6	0.403	0.256	0.267	0.279	0.060	1.984	0.280	0.436	0.216
NG099 ^a^	76.7	0.400	0.261	0.266	0.288	0.011	1.968	n/c	n/c	n/c
NG101 ^a^	79.1	0.405	0.259	0.270	0.282	0.047	2.012	0.248	0.396	0.187
NG103 ^a^	80.1	0.396	0.245	0.263	0.275	0.067	2.076	0.218	0.380	0.146
NG105 ^a^	78.6	0.395	0.259	0.263	0.274	0.057	1.971	0.249	0.386	0.195
NG106 ^a^	77.2	0.382	0.227	0.252	0.264	0.103	2.067	0.286	0.548	0.188
NG107 ^a^	75.7	0.402	0.246	0.270	0.282	0.086	1.959	0.286	0.496	0.201
NG108 ^a^	70.9	0.380	0.237	0.255	0.269	0.081	1.974	0.272	0.484	0.190
NG109 ^a^	79.6	0.408	0.244	0.272	0.284	0.115	2.031	0.306	0.465	0.242
NG111 ^a^	80.1	0.405	0.234	0.269	0.281	0.134	2.045	0.278	0.568	0.165
NG112 ^a^	79.1	0.392	0.233	0.259	0.271	0.110	1.940	0.295	0.532	0.203
NG113 ^a^	77.7	0.388	0.237	0.257	0.269	0.073	2.073	0.274	0.453	0.203
NG123 ^a^	82.5	0.414	0.256	0.275	0.288	0.090	2.008	0.283	0.462	0.214
NG124 ^b^	72.8	0.379	0.217	0.253	0.264	0.133	1.936	0.287	0.493	0.204
Fogera ^c^	80.1	0.407	0.253	0.270	0.283	0.072	1.969	0.300	0.519	0.213
Shambu ^c^	81.1	0.398	0.238	0.262	0.274	0.091	2.011	0.284	0.487	0.205
Mean_Alt-1	77.8	0.397	0.245	0.264	0.277	0.088	2.005	0.275	0.447	0.205
Mean_Alt-2	77.5	0.397	0.244	0.265	0.277	0.086	2.000	0.289	0.517	0.196
Mean_Alt-3	79.0	0.398	0.245	0.265	0.278	0.075	2.013	0.265	0.470	0.182
Mean_Reg-1	78.8	0.401	0.251	0.266	0.279	0.076	2.006	0.281	0.458	0.211
Mean_Reg-2	73.8	0.382	0.240	0.255	0.267	0.073	2.031	0.262	0.456	0.183
Mean_Reg-3	79.7	0.405	0.244	0.270	0.282	0.100	1.983	0.285	0.495	0.197
Mean_Reg-4	77.4	0.391	0.236	0.260	0.272	0.093	2.010	0.285	0.507	0.199
Mean_Reg-5	80.1	0.396	0.245	0.263	0.275	0.067	2.076	0.218	0.380	0.146
Mean_Reg-6	79.1	0.407	0.250	0.271	0.286	0.077	1.982	0.282	0.533	0.181
Mean_Landrace	78.1	0.397	0.245	0.264	0.277	0.083	2.006	0.277	0.478	0.195
Mean_Cultivar	80.6	0.4025	0.245	0.266	0.278	0.081	1.99	0.292	0.503	0.209
Mean_all	78.1	0.397	0.244	0.264	0.277	0.085	2.002	0.278	0.481	0.197
SE_all	0.006	0.004	0.003	0.003	0.003	0.006	0.050	0.005	0.011	0.004

Acc = accessions (^a^ landrace populations, ^b^ breeding population, ^c^ cultivars); PPL = percent polymorphic loci; I = Shannon’s information index; Ho = observed heterozygosity; He = expected heterozygosity; uHe = unbiased expected heterozygosity; F = fixation index; Theta (H) = Theta from mean heterozygosity under the stepwise mutation model [52]; EAED_1, 2, and 3 = estimates of average evolutionary divergence over sequence pairs within populations for (a) all polymorphic loci, (b) loci with allele frequency of equal or above 0.3 and below or equal 0.7, and (c) loci with allele frequency of below 0.3 and above 0.7, respectively, as estimated based on the Tajima-Nei model [53]. Note: In all cases, genotypes with more than 7% missing data were excluded. The Pearson correlation coefficient of EAED_1_ vs. EAED_2_, EAED_1_ vs. EAED_3_, and EAED_2_ vs. EAED_3_ were 0.79 (*p* < 0.001), 0.75 (*p* < 0.001), and 0.20 (*p* = 0.35). The 21 landrace accessions were grouped into three altitudinal groups and six regional groups (see Table S1).

**Table 3:** Analysis of molecular variance (AMOVA), based on 1023 permutations, for 24 accessions without grouping, and for 21 accessions by grouping them according to altitudinal range or regions of origin.

Source of Variation	DF	Sum of Squares	Variance Components	Percentage of Variation	Fixation Indices	Probability (*p*) Value
Among accessions	23	1078.2	1.019 Va	4.52	F_ST_ = 0.045	Va & F_ST_ < 0.0001
Among individuals within accessions	259	5921.0	1.321 Vb	5.86	F_IS_ = 0.061	Vb & F_IS_ < 0.0001
Within individuals	283	5722.0	20.219 Vc	89.63	F_IT_ = 0.104	Vc & F_IT_ < 0.0001
Total	565	12,721.3	22.556			
^a^ Among alt groups	2	97.9	0.026 Va	0.12	F_CT_ = 0.001	Va & F_CT_ = 0.1935
Among accessions within alt groups	18	800.1	1.015 Vb	4.67	F_SC_ = 0.047	Vb & F_SC_ < 0.0001
Within accessions	471	9746.7	20.694 Vc	95.21	F_ST_ = 0.048	Vc & F_ST_ < 0.0001
Total	491	10,644.8	21.735			
^b^ Among regions-I	5	217.0	−0.027 Va	−0.12	F_CT_ = −0.001	Va & F_CT_ = 0.6715
Among accessions within regions-I	15	681.0	1.056 Vb	4.86	F_SC_ = 0.048	Vb & F_SC_ < 0.0001
Within accessions	471	9746.7	20.694 Vc	95.26	F_ST_ = 0.047	Vc & F_ST_ < 0.0001
Total	491	10,644.8	21.723			
^c^ Among regions-II	1	55.4	0.044 Va	0.20	F_CT_ = 0.002	Va & F_CT_ = 0.047
Among accessions within regions-II	19	842.6	1.011 Vb	4.65	F_SC_ = 0.047	Vb & F_SC_ < 0.0001
Within accessions	471	9746.7	20.694 Vc	95.15	F_ST_ = 0.048	Vc & F_ST_ < 0.0001
Total	491	10,644.8	21.723			

Note: 21 of the 24 accessions were grouped according to region or altitudinal (alt) range or origin. The two cultivars and the breeding population were excluded from the grouping, as they cannot be placed in any of the groups. ^a^ The 21 accessions were grouped into three altitudinal groups: 1400–1680 m above sea level (masl), 1820–1968 masl, and 2045–2590 masl. ^b^ The 21 accessions were grouped into six regions (regions-I), and ^c^ the 21 accessions were grouped into two regions (regions-II) (Table S1).

**Table 4:** Analysis of molecular variance (AMOVA)-based pairwise F_ST_ between the 24 populations with 1023 permutations (below the diagonal) and mean F_ST_ of each accession against all other accessions (diagonal).

**Acc**	086	088	089	090	092	095	096	097	098	099	101	103	105	106	107	108	109	111	112	113	123	124	Fog	Sha
086	**0.036**																							
088	0.031	**0.041**																						
089	0.026	0.041	**0.045**																					
090	0.031	0.055	0.041	**0.052**																				
092	0.023	0.026	0.046	0.041	**0.038**																			
095	0.047	0.031	0.051	0.056	0.043	**0.052**																		
096	0.054	0.075	0.076	0.081	0.061	0.095	**0.074**																	
097	0.069	0.066	0.065	0.082	0.065	0.064	0.124	**0.076**																
098	0.046	0.061	0.037	0.073	0.037	0.074	0.079	0.08	**0.055**															
099	0.030	0.053	0.042	0.053	0.040	0.048	0.078	0.082	0.066	**0.048**														
101	0.034	0.032	0.053	0.065	0.050	0.064	0.08	0.078	0.057	0.075	**0.052**													
103	0.024	0.031	0.031	0.031	0.017	0.041	0.075	0.069	0.052	0.027	0.036	**0.035**												
105	0.034	0.033	0.043	0.045	0.042	0.046	0.069	0.084	0.049	0.054	0.048	0.038	**0.047**											
106	0.031	0.036	0.039	0.044	0.036	0.049	0.072	0.069	0.043	0.054	0.042	0.024	0.035	**0.043**										
107	0.023	0.039	0.039	0.033	0.033	0.043	0.069	0.078	0.047	0.034	0.042	0.025	0.035	0.035	**0.039**									
108	0.038	0.057	0.059	0.057	0.031	0.056	0.103	0.091	0.042	0.068	0.068	0.043	0.051	0.043	0.038	**0.056**								
109	0.050	0.037	0.050	0.061	0.040	0.05	0.072	0.064	0.048	0.059	0.047	0.029	0.056	0.05	0.054	0.068	**0.052**							
111	0.025	0.029	0.028	0.041	0.027	0.042	0.05	0.086	0.056	0.025	0.045	0.024	0.037	0.045	0.034	0.055	0.042	**0.039**						
112	0.034	0.046	0.053	0.049	0.039	0.045	0.072	0.089	0.05	0.037	0.057	0.047	0.041	0.046	0.031	0.063	0.06	0.043	**0.049**					
113	0.038	0.059	0.053	0.041	0.050	0.057	0.066	0.075	0.072	0.023	0.061	0.03	0.062	0.055	0.033	0.07	0.061	0.046	0.041	**0.051**				
123	0.026	0.007 *	0.020	0.039	0.018	0.044	0.057	0.055	0.034	0.037	0.027	0.019	0.021	0.025	0.023	0.043	0.047	0.006 *	0.037	0.043	**0.031**			
124	0.065	0.056	0.077	0.091	0.068	0.069	0.09	0.083	0.086	0.062	0.078	0.072	0.084	0.074	0.065	0.093	0.076	0.06	0.083	0.065	0.058	**0.072**		
Fog	0.028	0.019	0.040	0.042	0.025	0.033	0.055	0.058	0.038	0.031	0.026	0.021	0.046	0.032	0.031	0.038	0.043	0.028	0.031	0.035	0.011 *	0.038	**0.033**	
Sha	0.012 *	0.023	0.023	0.036	0.019	0.04	0.054	0.065	0.03	0.034	0.031	0.007 *	0.028	0.020	0.020	0.023	0.021	0.011 *	0.03	0.032	0.010 *	0.064	0.010 *	**0.028**

* = No significant differentiation between the pair of accessions. The first column and row are accession names without their two initial letters (*NG*) for the first 22 accessions. Acc = Accession; Fog = Fogera and Sha = Shambu. Bold: mean F_ST_ values of each accession against all other accessions.

## Supplementary Materials

The following are available online at https://www.mdpi.com/2073-4425/11/11/1373/s1, Table S1: Accession code and type, and altitude, region, and location of collecting sites, Table S2: GenBank accession numbers of the 628 ESTs containing 959 SNPs initially used in the development of the KASP markers, and grouping of the SNP loci according to the results of the KASP analysis, Table S3: Polymorphic information content (PIC), gene diversity (H), fixation indices, gene flow (Nm), observed (Ho) and expected (He) heterozygosity, and *p*-value for deviation of each locus from HWE, for the 202 polymorphic SNP loci, Table S4: Population size (N) and frequency of alleles (A, C, G, or T) at the 202 polymorphic loci for the three altitudinal groups (Alt-1, 2, 3) formed from the 21 landrace accessions.

## References

[1] Dagne K., Heneen W.K. (2008). The karyotype and nucleoli of *Guizotia abyssinica* (Compositae). Hereditas.

[2] Hiremath S.C., Murthy H.N. (1992). Cytological studies in *Guizotia* (Asteraceae). Caryologia.

[3] Dagne K. (1995). Karyotypes, C-banding and nucleolar numbers in *Guizotia* (Compositae). Plant Syst. Evol..

[4] Hiremath S.C., Murthy H.N., Salimath S.S. (1992). Quantitative nuclear DNA differences associated with genome evolution in *Guizotia* (Compositae). Genetica.

[5] Ohta T. (1994). Further Examples of Evolution by Gene Duplication Revealed through DNA Sequence Comparisons. Genetics.

[6] Meyerowitz E.M. (1999). The first completely sequenced plant chromosomes, from the mustard *Arabidopsis thaliana*, reveal a dynamic genome that is constantly being rearranged. Nature.

[7] Bennetzen J.L., Ma J., Devos K.M. (2005). Mechanisms of Recent Genome Size Variation in Flowering Plants. Ann. Bot..

[8] Weiss E.A. (1983). Oil Seed Crops.

[9] Murthy H.N., Hiremath S.C., Salimath S.S. (1993). Origin, evolution and genome differentiation in *Guizotia abyssinica* and its wild species. Theor. Appl. Genet..

[10] Getinet A., Sharma S.M. (1996). Niger (*Guizotia abyssinica* (L.F.)) Cass. Promoting the Conservation and Use of Underutilized and Neglected Crops.

[11] Geleta M., Ortiz R. (2013). The importance of *Guizotia abyssinica* (niger) for sustainable food security in Ethiopia. Genet. Resour. Crop. Evol..

[12] Geleta M., Stymne S., Bryngelsson T. (2011). Variation and inheritance of oil content and fatty acid composition in niger (*Guizotia abyssinica*). J. Food Compos. Anal..

[13] Dagne K., Jonsson A. (1997). Oil Content and Fatty Acid Composition of Seeds of *Guizotia* Cass (Compositae). J. Sci. Food Agric..

[14] Ramadan M.F., Ramadan M.F. (2012). Functional Properties, Nutritional Value, and Industrial Applications of Niger Oilseeds (*Guizotia abyssinica* Cass.). Crit. Rev. Food Sci. Nutr..

[15] Deme T., Haki G.D., Retta N., Woldegiorgis A.Z., Geleta M. (2017). Mineral and Anti-Nutritional Contents of Niger Seed (*Guizotia abyssinica* (L.f.) Cass., Linseed (*Linum usitatissimum* L.) and Sesame (*Sesamum indicum* L.) Varieties Grown in Ethiopia. Foods.

[16] Geleta M., Asfaw Z., Bekele E., Teshome A. (2002). Edible oil crops and their integration with the major cereals in North Shewa and South Welo, Central Highlands of Ethiopia: An ethnobotanical perspective. Hereditas.

[17] Alemaw G., Alamayehu N. (1997). Highland oilcrops: A two-decade research experience in Ethiopia. Research Report No. 30.

[18] Dempewolf H., Kane N.C., Ostevik K.L., Geleta M., Barker M.S., Lai Z., Stewart M.L., Bekele E., Engels J.M.M., Cronk Q.C.B. (2010). Establishing genomic tools and resources for *Guizotia abyssinica* (L.f.) Cass.-the development of a library of expressed sequence tags, microsatellite loci, and the sequencing of its chloroplast genome. Mol. Ecol. Resour..

[19] Geleta M., Bryngelsson T., Bekele E., Dagne K. (2006). Genetic diversity of *Guizotia abyssinica* (L.f.) Cass. (Asteraceae) from Ethiopia as revealed by random amplified polymorphic DNA (RAPD). Genet. Resour. Crop. Evol..

[20] Geleta M., Bryngelsson T., Bekele E., Dagne K. (2008). Assessment of genetic diversity of *Guizotia abyssinica* (L.f.) Cass. (Asteraceae) from Ethiopia using amplified fragment length polymorphism. Plant Genet. Resour..

[21] Petros Y., Merker A., Zeleke H. (2007). Analysis of genetic diversity of *Guizotia abyssinica* from Ethiopia using inter simple sequence repeat markers. Hereditas.

[22] Dempewolf H., Tesfaye M., Teshome A., Bjorkman A.D., Andrew R.L., Scascitelli M., Black S., Bekele E., Engels J.M.M., Cronk Q.C.B. (2015). Patterns of domestication in the Ethiopian oil-seed crop noug (*Guizotia abyssinica*). Evol. Appl..

[23] Ertiro B.T., Ogugo V., Worku M., Das B., Olsen M., Labuschagne M., Semagn K. (2015). Comparison of Kompetitive Allele Specific PCR (KASP) and genotyping by sequencing (GBS) for quality control analysis in maize. BMC Genom..

[24] Shi Z., Liu S., Noe J., Arelli P., Meksem K., Li Z. (2015). SNP identification and marker assay development for high-throughput selection of soybean cyst nematode resistance. BMC Genom..

[25] Kabbaj H., Sall A.T., Al-Abdallat A., Geleta M., Amri A., Filali-Maltouf A., Belkadi B., Ortiz R., Bassi F.M. (2017). Genetic Diversity within a Global Panel of Durum Wheat (*Triticum durum*) Landraces and Modern Germplasm Reveals the History of Alleles Exchange. Front. Plant Sci..

[26] Contreras-Soto R.I., Mora F., De Oliveira M.A.R., Higashi W., Scapim C.A., Schuster I. (2017). A Genome-Wide Association Study for Agronomic Traits in Soybean Using SNP Markers and SNP-Based Haplotype Analysis. PLoS ONE.

[27] Qu C., Jia L., Fu F., Zhao H., Lu K., Wei L., Xu X., Liang Y., Li S., Wang R. (2017). Genome-wide association mapping and Identification of candidate genes for fatty acid composition in *Brassica napus* L. using SNP markers. BMC Genom..

[28] Liu R., Gong J., Xiao X., Zhang Z., Li J., Liu A., Lu Q., Shang H., Shi Y., Ge Q. (2018). GWAS Analysis and QTL Identification of Fiber Quality Traits and Yield Components in Upland Cotton Using Enriched High-Density SNP Markers. Front. Plant Sci..

[29] Belaj A., De La Rosa R., Lorite I.J., Mariotti R., Cultrera N.G., Beuzón C.R., González-Plaza J.J., Muñoz-Merida A., Trelles O., Baldoni L. (2018). Usefulness of a New Large Set of High Throughput EST-SNP Markers as a Tool for Olive Germplasm Collection Management. Front. Plant Sci..

[30] Kim B., Hwang I.S., Lee H.J., Lee J.M., Seo E., Choi D., Oh C.-S. (2018). Identification of a molecular marker tightly linked to bacterial wilt resistance in tomato by genome-wide SNP analysis. Theor. Appl. Genet..

[31] Geleta M., Gustafsson C., Glaubitz J.C., Ortiz R. (2020). High-Density Genetic Linkage Mapping of *Lepidium* Based on Genotyping-by-Sequencing SNPs and Segregating Contig Tag Haplotypes. Front. Plant Sci..

[32] Palmé A.E., Hagenblad J., Solberg S.Ø., Aloisi K., Artemyeva A.M. (2020). SNP Markers and Evaluation of Duplicate Holdings of *Brassica oleracea* in Two European Genebanks. Plants.

[33] Zanotto S., Vandenberg A., Khazaei H. (2019). Development and validation of a robust KASP marker for zt2 locus in faba bean (*Vicia faba*). Plant Breed..

[34] Islam A.S.M.F., Blair M.W. (2018). Molecular Characterization of Mung Bean Germplasm from the USDA Core Collection Using Newly Developed KASP-based SNP Markers. Crop. Sci..

[35] Grimm K.D.S., Porter L.D. (2020). Development and Validation of KASP Markers for the Identification of Pea seedborne mosaic virus Pathotype P1 Resistance in *Pisum sativum*. Plant Dis..

[36] Leal-Bertioli S.C.M., Cavalcante U., Gouveia E.G., Ballén-Taborda C., Shirasawa K., Guimarães P.M., Jackson S.A., Bertioli D.J., Moretzsohn M.C. (2015). Identification of QTLs for Rust Resistance in the Peanut Wild Species *Arachis magna* and the Development of KASP Markers for Marker-Assisted Selection. G3 Genes. Genom. Genet..

[37] Kang J.-W., Lee S.-B., Lee J.-Y., Kwon Y.-H., Lee S.-M., Rolly N.K., Shin D., Cha J.-G., Park D.-S., Ko J.-M. (2020). Development and Validation of KASP Markers for *Stv-bi*, a Rice Stripe Virus Resistance Gene in Rice (*Oryza sativa* L.). Plant Breed. Biotechnol..

[38] Cheon K.-S., Baek J., Cho Y.-I., Jeong Y.-M., Lee Y.-Y., Oh J., Won Y.J., Kang D.-Y., Oh H., Kim S.L. (2018). Single Nucleotide Polymorphism (SNP) Discovery and Kompetitive Allele-Specific PCR (KASP) Marker Development with Korean Japonica Rice Varieties. Plant Breed. Biotechnol..

[39] Han G., Liu S., Jin Y., Jia M., Ma P., Liu H., Wang J., An D.-G. (2020). Scale development and utilization of universal PCR-based and high-throughput KASP markers specific for chromosome arms of rye (*Secale cereale* L.). BMC Genom..

[40] Burow G., Chopra R., Sattler S., Burke J., Acosta-Martinez V., Xin Z. (2019). Deployment of SNP (CAPS and KASP) markers for allelic discrimination and easy access to functional variants for brown midrib genes *bmr6* and *bmr12* in *Sorghum bicolor*. Mol. Breed..

[41] Wu J., Wang Q., Kang Z., Liu S., Li H., Mu J., Dai M., Han D., Zeng Q., Chen X. (2017). Development and Validation of KASP-SNP Markers for QTL Underlying Resistance to Stripe Rust in Common Wheat Cultivar P10057. Plant Dis..

[42] Fang T., Lei L., Li G., Powers C., Hunger R.M., Carver B.F., Yan L. (2020). Development and deployment of KASP markers for multiple alleles of Lr34 in wheat. Theor. Appl. Genet..

[43] Makhoul M., Rambla C., Voss-Fels K.P., Hickey L.T., Snowdon R.J., Obermeier C. (2020). Overcoming polyploidy pitfalls: A user guide for effective SNP conversion into KASP markers in wheat. Theor. Appl. Genet..

[44] Geleta M., Bryngelsson T. (2010). Population genetics of self-incompatibility and developing self-compatible genotypes in *Guizotia abyssinica* (L.f.) Cass. (Asteraceae). Euphytica.

[45] Hodgins K.A., Lai Z., De Oliveira L.O., Still D.W., Scascitelli M., Barker M.S., Kane N.C., Dempewolf H., Kozik A., Kesseli R.V. (2013). Genomics of Compositae crops: Reference transcriptome assemblies and evidence of hybridization with wild relatives. Mol. Ecol. Resour..

[46] Li H., Durbin R. (2009). Fast and accurate short read alignment with Burrows-Wheeler transform. Bioinformatics.

[47] Li H., Handsaker B., Wysoker A., Fennell T., Ruan J., Homer N., Marth G., Abecasis G., Durbin R., Subgroup G.P.D.P. (2009). The sequence alignment/map format and SAMtools. Bioinformatics.

[48] Yeh F., Yang R., Boyle T. (1999). POPGENE: Microsoft Window-Based Freeware for Population Genetic Analysis.

[49] Peakall R., Smouse P.E. (2012). GenAlEx 6.5: Genetic analysis in Excel. Population genetic software for teaching and research--an update. Bioinformatics.

[50] Excoffier L., Lischer H.E.L. (2010). Arlequin suite ver 3.5: A new series of programs to perform population genetics analyses under Linux and Windows. Mol. Ecol. Resour..

[51] Kumar S., Stecher G., Tamura K.K. (2016). MEGA7: Molecular evolutionary genetics analysis version 7.0 for bigger datasets. Mol. Biol. Evol..

[52] Nei M. (1987). Molecular Evolutionary Genetics.

[53] Tajima F., Nei M. (1984). Estimation of evolutionary distance between nucleotide sequences. Mol. Biol. Evol..

[54] Pritchard J.K., Stephens M., Donnelly P. (2000). Inference of population structure using multilocus genotype data. Genetics.

[55] Li Y.-L., Liu J.-X. (2017). StructureSelector: A web-based software to select and visualize the optimal number of clusters using multiple methods. Mol. Ecol. Resour..

[56] Evanno G., Regnaut S., Goudet J. (2005). Detecting the number of clusters of individuals using the software structure: A simulation study. Mol. Ecol..

[57] Kopelman N.M., Mayzel J., Jakobsson M., Rosenberg N.A., Mayrose I. (2015). Clumpak: A program for identifying clustering modes and packaging population structure inferences across K. Mol. Ecol. Resour..

[58] Moragues M., Comadran J., Waugh R., Milne I., Flavell A.J., Russell J.R. (2010). Effects of ascertainment bias and marker number on estimations of barley diversity from high-throughput SNP genotype data. Theor. Appl. Genet..

[59] Nielsen R. (2000). Estimation of population parameters and recombination rates from single nucleotide polymorphisms. Genetics.

[60] Chang H.-W., Cheng Y.-H., Chuang L.-Y., Yang C.-H. (2010). SNP-RFLPing 2: An updated and integrated PCR-RFLP tool for SNP genotyping. BMC Bioinform..

[61] Evans J., Kim J., Childs K.L., Vaillancourt B., Crisovan E., Nandety A., Gerhardt D.J., Richmond T.A., Jeddeloh J.A., Kaeppler S.M. (2014). Nucleotide polymorphism and copy number variant detection using exome capture and next-generation sequencing in the polyploid grass *Panicum virgatum*. Plant J..

[62] Hildebrand C.E., Torney D.V., Wagner R.P. (1994). Informativeness of polymorphic DNA markers. The Human Genome Project: Decipering the Blueprint of Heredity (Cooper NG edited).

[63] Shete S., Tiwari H., Elston R.C. (2000). On Estimating the Heterozygosity and Polymorphism Information Content Value. Theor. Popul. Biol..

[64] Singh N., Choudhury D.R., Singh A.K., Kumar S., Srinivasan K., Tyagi R.K., Singh R. (2013). Comparison of SSR and SNP Markers in Estimation of Genetic Diversity and Population Structure of Indian Rice Varieties. PLoS ONE.

[65] Chen H., He H., Zou Y., Chen W., Yu R., Liu X., Yang Y., Gao Y.-M., Xu J.-L., Fan L.-M. (2011). Development and application of a set of breeder-friendly SNP markers for genetic analyses and molecular breeding of rice (*Oryza sativa* L.). Theor. Appl. Genet..

[66] Geleta M., Bryngelsson T., Bekele E., Dagne K. (2007). AFLP and RAPD analyses of genetic diversity of wild and/or weedy *Guizotia* (Asteraceae) from Ethiopia. Hereditas.

[67] Milligan B.G., McMurry C.K. (1993). Dominant vs. codominant genetic markers in the estimation of male mating success. Mol. Ecol..

[68] Bonnett D., Rebetzke G., Spielmeyer W. (2005). Strategies for efficient implementation of molecular markers in wheat breeding. Mol. Breed..

[69] Foll M., Gaggiotti O.E. (2008). A Genome-Scan Method to Identify Selected Loci Appropriate for Both Dominant and Codominant Markers: A Bayesian Perspective. Genetics.

[70] Wright S. (1949). The genetical structure of populations. Ann. Eugen..

[71] Wright S. (1965). The Interpretation of Population Structure by F-Statistics with Special Regard to Systems of Mating. Evolution.

[72] Nemomissa S., Bekele E., Dagne K. (1999). Self-incompatibility system in the Ethiopian populations of *Guizotia abyssinica* (L.F.) Cass. (niger). SINET Ethiop. J. Sci..

[73] Waters E.R. (1995). The Molecular Evolution of the Small Heat-Shock Proteins in Plants. Genetics.

[74] Hamrick J.L., Godt M.J.W. (1996). Effects of life history traits on genetic diversity in plant species. Philos. Trans. R. Soc. B Biol. Sci..

[75] Nybom H. (2004). Comparison of different nuclear DNA markers for estimating intraspecific genetic diversity in plants. Mol. Ecol..

[76] Gadissa F., Tesfaye K., Dagne K., Geleta M. (2018). Genetic diversity and population structure analyses of *Plectranthus edulis* (Vatke) Agnew collections from diverse agro-ecologies in Ethiopia using newly developed EST-SSRs marker system. BMC Genet..

[77] Ng’uni D., Geleta M., Hofvander P., Fatih M., Bryngelsson T. (2012). Comparative genetic diversity and nutritional quality variation among some important Southern African sorghum accessions [*Sorghum bicolor* (L.) Moench]. Aust. J. Crop. Sci..

[78] Ng’Uni D., Geleta M., Bryngelsson T. (2011). Genetic diversity in sorghum (*Sorghum bicolor* (L.) Moench) accessions of Zambia as revealed by simple sequence repeats (SSR). Hereditas.

[79] Motlhaodi T., Geleta M., Bryngelsson T., Fatih M., Chite S., Ortiz R. (2014). Genetic diversity in ex-situ conserved sorghum accessions of Botswana as estimated by microsatellite markers. Aust. J. Crop. Sci..

[80] Motlhaodi T., Geleta M., Chite S., Fatih M., Ortiz R., Bryngelsson T. (2017). Genetic diversity in sorghum germplasm from Southern Africa as revealed by microsatellite markers and agromorphological traits. Genet. Resour. Crop. Evol..

[81] Hegay S., Geleta M., Bryngelsson T., Gustavsson L., Hovmalm H.P., Ortiz R. (2012). Comparing genetic diversity and population structure of common beans grown in Kyrgyzstan using microsatellites. Sci. J. Crop. Sci..

[82] Teshome A., Bryngelsson T., Dagne K., Geleta M. (2015). Assessment of genetic diversity in Ethiopian field pea (*Pisum sativum* L.) accessions with newly developed EST-SSR markers. BMC Genet..

[83] Geleta M., Herrera I., Monzón A., Bryngelsson T. (2012). Genetic Diversity of Arabica Coffee (*Coffea arabica* L.) in Nicaragua as Estimated by Simple Sequence Repeat Markers. Sci. World J..

[84] Chombe D., Bekele E., Bryngelsson T., Teshome A., Geleta M. (2017). Genetic structure and relationships within and between cultivated and wild korarima [*Aframomum corrorima* (Braun) P.C.M. Jansen] in Ethiopia as revealed by simple sequence repeat (SSR) markers. BMC Genet..

[85] Wright S. (1943). Isolation by Distance. Genetics.

[86] Ishikawa M., Naito S., Ohno T. (1993). Effects of the *tom1* mutation of *Arabidopsis thaliana* on the multiplication of tobacco mosaic virus RNA in protoplasts. J. Virol..

[87] Yamanaka T., Ohta T., Takahashi M., Meshi T., Schmidt R., Dean C., Naito S., Ishikawa M. (2000). *TOM1*, an *Arabidopsis* gene required for efficient multiplication of a tobamovirus, encodes a putative transmembrane protein. Proc. Natl. Acad. Sci. USA.

[88] Rannala B., Mountain J., Rannala B., Mountain J.L. (1997). Detecting immigration by using multilocus genotypes. Proc. Natl. Acad. Sci. USA.

[89] Davies N., Villablanca F.X., Roderick G.K. (1999). Determining the source of individuals: Multilocus genotyping in nonequilibrium population genetics. Tree.

[90] Alexander D.H., Novembre J., Lange K. (2009). Fast model-based estimation of ancestry in unrelated individuals. Genome Res..

